# The Plane Tree of Hippocrates

**Published:** 1906-08-18

**Authors:** 


### THE PLANE TREE OF HIPPOCRATES.

The Royal College of Surgeons of England has lately received from Dr. Edward Clapton a present which is as unusual as it is interesting. Dr. Clapton has presented two pieces of branch, a bundle of twigs from the branches, and a small box of leaves and round button-like catkins of the celebrated plane tree at Cos, under the shade of whose spreading boughs it is traditionally reported that Hippocrates taught medicine more than 2,300 years ago. Whatever the exact truth may be, the tree has undoubtedly great sentimental interest attaching to it. Good care has always been taken of it, as is shown by the way in which its branches are supported by pieces of antique columns, a podium or raised platform has been built round its trunk, and we are told that the Sultan attaches so great an importance to its preservation that a guard is posted over it day and night. Close by the tree is a solid marble seat, which is said to be the chair of Hippocrates. The tree is situated about two minutes' walk from the landing-stage, in a small open place, bounded on one side by the mediæval castle of Cos, and on the other by a church formerly Christian, but now turned into a mosque, and a few coffee-houses. Close to the plane tree is a handsome public fountain, the cupola of which is supported by columns. The tree itself is undoubtedly of great age. It measures 57 feet in circumference, and, though the trunk is hollow, and the large middle branches of the crown are broken away, the lower branches are still well preserved, and one of them especially is very long, and spreads horizontally. The tree is so old, and the tradition connecting it with the father of medicine is so definite, that we may well believe Dean Farrar's statement, in the Life and Work of St. Paul, that "neither the wines nor the purple nor the perfumes of Cos would have much interest for the little band (attending St. Paul and St. Luke, who spent one day in the island); but, if opportunity offered, we may be sure that 'the beloved physician' would not miss the opportunity of seeing all that he could of the scientific memorials of the Asclepiads—the great medical school of the ancient world."

The plane tree has always been held in high repute throughout the world, as it still is with ourselves in London, where it flourishes because it is capable of shedding its bark, and by so doing gets rid of the soot which might otherwise clog its growth. Ezekiel, in enumerating the trees in the Garden of God, enumerates the cedar, the fir, and the plane. The Greeks planted the tree on a large scale, and in the shady groves of Academe the Greek youths received their lessons in philosophy.

